# Periodic Nonlinear Error Analysis and Compensation of a Single-Excited Petal-Shaped Capacitive Encoder to Achieve High-Accuracy Measurement

**DOI:** 10.3390/s19102412

**Published:** 2019-05-27

**Authors:** Bo Hou, Bin Zhou, Xiang Li, Bowen Xing, Luying Yi, Qi Wei, Rong Zhang

**Affiliations:** Engineering Research Center for Navigation Technology, Department of Precision Instrument, Tsinghua University, Beijing 100084, China; houb15@mails.tsinghua.edu.cn (B.H.); li-x07@mails.tsinghua.edu.cn (X.L.); xingbw17@mails.tsinghua.edu.cn (B.X.); yily15@mails.tsinghua.edu.cn (L.Y.)

**Keywords:** capacitance encoder, single-excitation, nonlinear error analysis, harmonic components compensation

## Abstract

The measurement results of a single-excitation petal-shaped capacitive encoder show strong periodic characteristics for nonlinear errors. This paper presents the analysis of periodic nonlinear errors in a single-excitation petal-shaped encoder in terms of three main aspects—sensitive structure processing error, circuit demodulation error, and installation error. Analytical and simulation results confirm that the first-, second-, and fourth-periodic electrical errors are caused by the misalignment of circuit parameters, non-uniform segmentation of the processing error, and cross interference of the electric field, respectively. Further experimental investigation reveals that the mechanical periodic error is caused by installation misalignment. Based on these analytical, simulation, and experimental results, the design of the capacitive encoder was optimized and a method based on harmonic components was applied to compensate the periodic nonlinear error of the encoder. Measurement results shows that the prototype which has 180 petal-shaped numbers can achieve a reduction of periodic nonlinear errors to less than 0.02° and its accuracy can be improved to 0.0006° after compensation over the full measurement range.

## 1. Introduction

Rotary encoders are widely used to provide positional feedback information and achieve precise control [1,2,3,4,5]. The encoders are classified as optical, magnetic, inductive, and capacitive according to their measurement principle. Optical and magnetic encoders are dominant in the market as they can achieve high precision, but they tend to be relatively expensive and bulky [2,6,7,8,9,10]. Currently, capacitive encoders have been gaining interest owing to their simplified design, potential for further miniaturization, insensitivity to magnetic field variation, long lifespan, low cost, and high measurement accuracy [5,10,11,12].

Capacitive rotary sensors achieve angle information encoding via various electrodes between rotor and stator. While rotating, capacitances formed between plates change and the angle of shaft can be obtained. Many types of capacitive encoders have been proposed, and the most prominent is by modulating the angular information to two orthogonal triangular signals [6,7,8]. Encoders of this type have the advantage of high robustness to common mode errors. Literature Refs. [6,7] have developed such kinds of angle encoders and achieved a measuring accuracy of 0.5°. However, the encoders shown in Refs. [6,7] have difficulty in achieving high measurement resolution and accuracy as they encode the mechanical angle to one electric period. To improve resolution and accuracy, multi-petal structures (similar to multi-pair resolvers) which can subdivide the mechanical angle, have been developed. In Ref. [2], a multi-petal structure with a nonlinear error less than 0.5° and a resolution up to 0.0008° was presented. The measurement result of nonlinear error shows great periodicity and limits measurement accuracy. In Ref. [13], a single-excited petal-shaped capacitive rotary encoder was proposed and achieved a measurement accuracy of 0.008°, but periodic nonlinear error also exists and the analysis of the encoder is insufficient.

Although the repeatability and periodicity of the nonlinear error results reported in Ref. [13] are excellent, they provide no systematic analysis about the cause of nonlinear error. To further improve measurement accuracy of the petal-shaped capacitive rotary encoder, this paper focused on three aspects of the single-excited petal-shaped capacitive encoder—its sensitive structure processing, demodulation circuit, and installation error—to distinguish the contribution to the periodic nonlinear error. Based on the analytical, simulation, and experimental results, an optimized redesign was carried out to reduce nonlinear error and a method based on the harmonic components was applied to compensate the residual periodic nonlinear error.

The remainder of this paper is organized as follows. We first present the basic operational and principles of the single-excited petal-shaped capacitive rotary encoder in Section 2. In Section 3, periodic nonlinear error analysis is conducted on three aspects; processing, circuit, and installation. In Section 4, the conclusion of nonlinear error analysis is presented. The experimental setup, measurement results and compensation are presented and discussed in Section 5. Finally, in Section 6 we provide a brief summary and conclusion.

## 2. Basic Principle and Design

**Figure 1:**
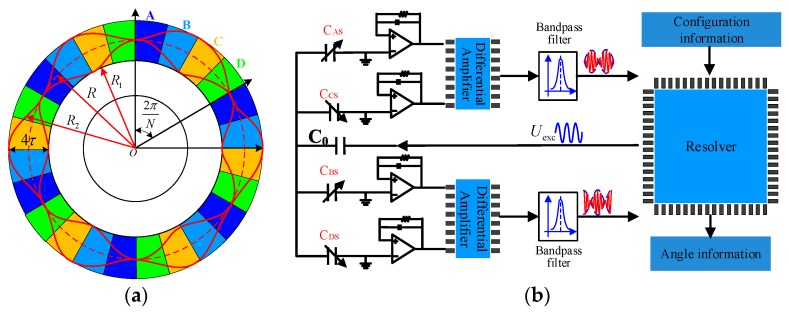
(**a**) Schematic of the proposed capacitive encoder, the red line represents the sensitive electrode and the colored sectors represent four collection electrodes; (**b**) circuit model diagram, including four C-V (capacitance-to-voltage) conversion modules, two differential amplifiers, two band pass filters, and a resolver chip.

A schematic and circuit model of the signal excited petal-shaped capacitive rotary encoder is shown in Figure 1a,b [13,14]. The red line represents the sensitive electrode on the rotor and the colored sectors represent four collection electrodes on the stator. The sensitive electrode of the rotor is comprised of a sinusoidal petal-shape. The contour lines of the sensitive electrode are given as

(1) $$\left\{ \begin{array}{l} R_{1}=(R-\tau )-\tau \cdot \cos(N\cdot \phi )=R-\tau -\tau \cdot \cos(\theta )\\ R_{2}=(R+\tau )+\tau \cdot \cos(N\cdot \phi )=R+\tau +\tau \cdot \cos(\theta ) \end{array}\right.$$

where $R_{1}$ and $R_{2}$ are the inner and the outer contour lines, respectively, R is the radius of the circle along which the sine wave is wrapped, *τ* is the sine wave amplitude, *N* is a positive integer representing the number of petal shapes, ϕ is the mechanical angle between the rotor and stator, θ is the electrical angle, which is *N* times the mechanical angle (θ=N⋅ϕ). The petal-shaped sensitive electrode can effectively subdivide the mechanical periodic to achieve a high precision measurement.

According to the capacitive coupling principle, the capacitances between the sensitive electrode on the rotor and the four collection electrodes on the stator can be obtained and presented as.

(2) $$\left\{ \begin{array}{l} C_{AS}=\frac{\varepsilon_{0}\varepsilon }{d}S_{AS}=\frac{\varepsilon_{0}\varepsilon }{d}\cdot \int_{(-\pi /4+\theta )/N}^{(\pi /4+\theta )/N}\frac{1}{2}({R_{2}}^{2}-{R_{1}}^{2})d\theta =\frac{4\varepsilon \varepsilon_{0}R\tau }{d}+\frac{4\varepsilon \varepsilon_{0}R\tau \cdot \sin\theta }{d}=C_{0}+\Delta C_{\sin(\theta )}\\ C_{BS}=\frac{\varepsilon_{0}\varepsilon }{d}S_{BS}=\frac{\varepsilon_{0}\varepsilon }{d}\cdot \int_{(-\pi /4+\theta )/N}^{(\pi /4+\theta )/N}\frac{1}{2}({R_{2}}^{2}-{R_{1}}^{2})d\theta =\frac{4\varepsilon \varepsilon_{0}R\tau }{d}+\frac{4\varepsilon \varepsilon_{0}R\tau \cdot \cos\theta }{d}=C_{0}+\Delta C_{\cos(\theta )}\\ C_{CS}=\frac{\varepsilon_{0}\varepsilon }{d}S_{CS}=\frac{\varepsilon_{0}\varepsilon }{d}\cdot \int_{(-\pi /4+\pi +\theta )/N}^{(\pi /4+\pi +\theta )/N}\frac{1}{2}({R_{2}}^{2}-{R_{1}}^{2})d\theta =\frac{4\varepsilon \varepsilon_{0}R\tau }{d}-\frac{4\varepsilon \varepsilon_{0}R\tau \cdot \sin\theta }{d}=C_{0}-\Delta C_{\sin(\theta )}\\ C_{DS}=\frac{\varepsilon_{0}\varepsilon }{d}S_{DS}=\frac{\varepsilon_{0}\varepsilon }{d}\cdot \int_{(-\pi /4+\pi +\theta )/N}^{(\pi /4+\pi +\theta )/N}\frac{1}{2}({R_{2}}^{2}-{R_{1}}^{2})d\theta =\frac{4\varepsilon \varepsilon_{0}R\tau }{d}-\frac{4\varepsilon \varepsilon_{0}R\tau \cdot \cos\theta }{d}=C_{0}-\Delta C_{\cos(\theta )} \end{array}\right.$$

where $S_{AS}$, $S_{BS}$, $S_{CS}$ and $S_{DS}$ represent the face area of sensitive electrode and collection electrodes,$C_{0}=4\varepsilon \varepsilon_{0}R\tau /d$ is the *DC* component, d is the distance of stator and rotor $\Delta C_{\sin(\theta )}=4\varepsilon \varepsilon_{0}R\tau \cdot \sin\theta /d$, and $\Delta C_{\cos(\theta )}=4\varepsilon \varepsilon_{0}R\tau \cdot \cos\theta /d$ is the angle encoded component.

Four charge amplifiers (C-V conversion modules) are applied to convert the four measurement capacitance changes to the voltages $U_{NAS}$, $U_{NBS}$, $U_{NCS}$, and $U_{NDS}$ as shown in Figure 1b.

Two differential amplifiers are used to eliminate the common element in Equation (2), producing two amplitude-modulated signals, $U_{NACS}$ and $U_{NBDS}$:

(3) $$U_{NACS}=U_{NAS}-U_{NCS}=2k\cdot \Delta C_{\sin(\theta )}\cdot \cos(\theta )=U_{S}\cdot \sin(N\cdot \phi )$$

(4) $$U_{NBDS}=U_{NBS}-U_{NDS}=2k\cdot \Delta C_{\cos(\theta )}\cdot \sin(\theta )=U_{C}\cdot \cos(N\cdot \phi )$$

where *k* is the amplification factor of the differential amplifier, $U_{C}=U_{S}=8\varepsilon \varepsilon_{0}R\tau k/d$ is the amplitude of the signal. It is seen from Equations (3) and (4) that the rotational mechanical angle, ϕ=θ/N, is encoded into signals $U_{NACS}$ and $U_{NBDS}$. As the number of petal forms increases, the angle can be further subdivided, improving the scale factor. However, *N* cannot be increased infinitely because the manufacturing error grows with *N*.

A resolver chip is used to decode the amplitude-modulated signal from *U_NACS_* and $U_{NBDS}$ to get the angular position [7,15,16]. The angle can be obtained as,

(5) β=arctan(UNACSUNBDS)=arctan(sin(N⋅ϕ)cos(N⋅ϕ))=arctan(sinθcosθ)

**Figure 2:**
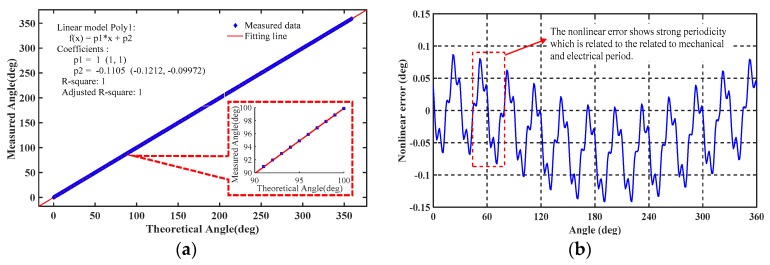
(**a**) Measured angular position and its best-fit line; (**b**) nonlinearity error with respect to best-fit line over the full measurement range.

As an effect of the various errors, including sensitive structure processing, circuit demodulation, and installation error, periodic nonlinearity errors including periodic electrical and mechanical errors occur in the measured output angle, as shown in Figure 2a,b. These nonlinear errors exhibit a strong periodic characteristic. To further improve the measurement accuracy and effectively compensate for these periodic errors, it is necessary to determine the cause of the nonlinear error and provide a corresponding solution. The following section presents a detailed analysis of the causes of periodic nonlinear error in a single-excitation capacitive encoder as a reference to further improve its accuracy.

## 3. Periodic Nonlinear Error Analysis

According to measurement principles, the nonlinear error is closely related to the quality of the orthogonal triangular signal, which is effected by various factors including the manufacturing and circuit error, the installation conditions, and the interference.

### 3.1. Processing Error Analyses

#### 3.1.1. Segmentation Error of the Collection Electrode

**Figure 3:**
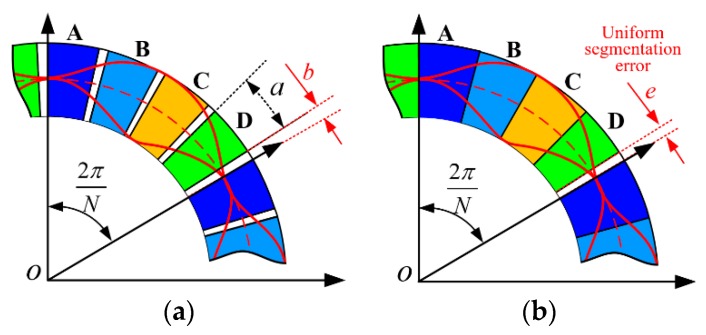
Schematic of collection electrodes with different machining error types. (**a**). Uniform segmentation error of collection electrodes; (**b**) non-uniform segmentation error and an error width of *e* deg.

The processing error is critical for the measurement accuracy, especially on the collection electrode of the stator. There are two kinds of typical processing error for the collection electrode. Figure 3a shows an example of uniform segmentation error of the collection electrode in which the widths of four electrodes and separation widths of each collection electrode are equal and represented as *a* and *b*. Figure 3b shows a case of non-uniform segmentation error, in which the non-uniform error is represented as *e* [17].

In the ideal case, the separation width *b* is approximately equal to zero. Due to the limitation of the PCB processing technology, the separation width *b* can only be as small as 4 mil (1 mil = 1/1000 inch = 0.0254 mm). When *N*, the number of petal shapes, is small, *a* is much larger than *b* and the error of *b* can be neglected. However, high-precision measurement requires large values of *N* for which the size of *b* cannot be ignored and it is necessary to analyze its influence on the final measurement result. The effect of *b* in the output capacitance value can be calculated using the following integrations:

(6) $$\left\{ \begin{array}{l} S_{AS}=\int_{(-\pi /4+\theta +b)/N}^{(\pi /4+\theta -b)/N}\frac{1}{2}({R_{2}}^{2}-{R_{1}}^{2})d\theta \\ S_{BS}=\int_{(-\pi /4+\theta +b)/N}^{(\pi /4+\theta -b)/N}\frac{1}{2}({R_{2}}^{2}-{R_{1}}^{2})d\theta \\ S_{CS}=\int_{(-\pi /4+\pi +\theta +b)/N}^{(\pi /4+\pi +\theta -b)/N}\frac{1}{2}({R_{2}}^{2}-{R_{1}}^{2})d\theta \\ S_{DS}=\int_{(-\pi /4+\pi +\theta +b)/N}^{(\pi /4+\pi +\theta -b)/N}\frac{1}{2}({R_{2}}^{2}-{R_{1}}^{2})d\theta \end{array}\right.$$

Referring to Equation (2), the relationship of the area to the angle of rotation can be expressed as

(7) $$\begin{array}{l} U_{NACS}=4R\tau (\sqrt{2}-2\cos(b))\times \sin\theta \\ U_{NBDS}=4R\tau (\sqrt{2}-2\cos(b))\times \cos\theta \end{array}$$

According to the demodulation principle, the final measured angle output in the presence of the uniform splitting error can then be expressed as

(8) $$\beta =\arctan(\frac{A\cdot 4R\tau (\sqrt{2}-2\cos(b))\times \sin\theta }{A\cdot 4R\tau (\sqrt{2}-2\cos(b))\times \cos\theta })=\arctan(\frac{\sin\theta }{\cos\theta })$$

It is seen from Equations (7) and (8) that the separation widths *b* has a great effect on the amplitude of the two output orthogonal signals and the amplitude is proportionally decreased. However, the separation width *b* does not affect the measurement accuracy in the final angle in the ideal situation, as the angle information is modulated into two orthogonal signals.

Non-uniform segmentation error is another possible source of collection electrode processing error. A special case is shown in Figure 3b, in which the non-uniform error *e* resulting in face error can be expressed as follows:

(9) $$\Delta S_{AS}=\int_{\phi }^{\left(\phi +e\right)}\frac{1}{2}({\left((R+\tau )+\tau \cos(N\phi )\right)}^{2}-{\left((R-\tau )-\tau \cos(N\phi )\right)}^{2})d\phi$$

Referring to Equation (2), the relationship of the area to the angle of rotation can be expressed as

(10) $$\begin{array}{l} U_{NACS}=U_{S}(\sin\theta +\frac{2R\tau \left(eN-\sin\left[N\phi \right]+\sin\left[N\left(e+\phi \right)\right]\right)}{N})\\ U_{NBDS}=U_{C}\cdot \cos(\theta ) \end{array}$$

According to the demodulation principle, the final measured angle output in the presence of the uniform splitting error can be expressed as

(11) $$\beta =\arctan\frac{A\sin(\theta )+\frac{2R\tau \left(eN-\sin\left[N\phi \right]+\sin\left[N\left(e+\phi \right)\right]\right)}{N}}{A\cos(\theta )}$$

**Figure 4:**
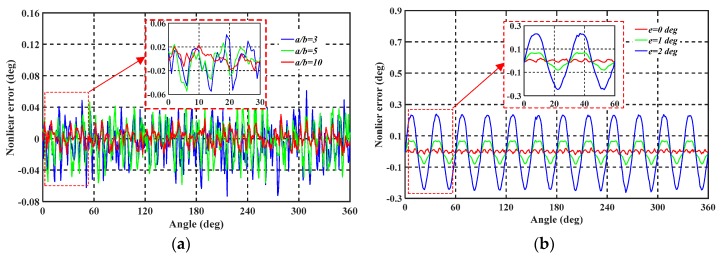
MATLAB simulation results of collection electrode segmentation error. (**a**) Angular output for a uniform segmentation error. (**b**) Angular output for a non-uniform splitting error, with blue, green, and red curves representing different non-uniform errors *e*.

We simulated the uniform and non-uniform machining error using COMSOL software to calculate the change of the sensitive capacitors. The result for the capacitor was then converted into the output angle β using MATLAB Simulink tools. In the simulation, the number of petal shapes *N* was set to 12. The simulation results are shown in Figure 4. Figure 4a shows the electrical angle output at different electrode widths *b*, with results that are consistent with the theoretical results obtained using Equation (8). As a uniform segmentation error on the collection electrode does not affect the measurement results, it is in theory possible to increase the number of subdivisions of the electrical period infinitely, and it is only necessary to ensure the separate widths *b* are uniformly distributed during the processing. However, a fourth-order error will occur as a result of electric field interference which is analyzed in the next section. Figure 4b shows the results of the same simulation for a non-uniform segmentation error of the collection electrode, from which it is seen that a non-uniform processing error will cause a periodic nonlinear error at the same frequency as the electrical periodic. The magnitude of the nonlinear error corresponds to non-uniformity of the collection electrode. The non-uniform error *e* must be effectively reduced during processing.

#### 3.1.2. Harmonic Components of Electric Field Interference between the Collection Electrodes

Due to electric field interference between the collection electrodes, the output quadrature signal may contain other harmonic components [18]. The measured signal can be expressed as:

(12) $$U_{S}=A\sin\theta +\sum_{n=1}^{\infty }A_{Sn}\sin n(\theta +\delta_{n}),U_{C}=A\cos\theta +\sum_{n=1}^{\infty }A_{Cn}\cos n(\theta +\eta_{n})$$

where *n* is the harmonic number, $A_{Sn}$ and $A_{Cn}$ are the amplitude-proportional coefficients, and $\delta_{n}$ and $\eta_{n}$ are the phases of the other harmonic components [19,20]. The amplitude modulation signals can be expressed as the following Fourier series:

(13) $$\begin{array}{l} U_{S}=A\sin\theta +A_{S2}\sin(2\theta )+A_{S3}\sin(3\theta )+A_{S4}\sin(4\theta )\cdots \\ U_{C}=A\cos\theta +A_{C2}\cos(2\theta )+A_{C3}\cos(3\theta )+A_{C4}\cos(4\theta )\cdots \end{array}$$

Because there are four electrodes per electric period and the four output voltages of the C-V model are subtracted in a single-excitation, there should be very little interference from the even-numbered frequency components in Equation (13), which can be omitted to simplify the calculation:

(14) $$U_{S}=\sin\theta \pm A_{S3}\sin3\theta \pm A_{S5}\sin5\theta \cdots \;U_{C}=\cos\theta \pm A_{C3}\cos3\theta \pm A_{C5}\cos5\theta \cdots$$

It is important to note that only odd harmonic components are present in the space domain. Normally, $1\geq A_{S3}=A_{C3}>A_{S5}=A_{C5}>A_{Si}=A_{Ci}\cdots$ and, for convenience of analysis, only the third-harmonic component in *U_S_* is taken into consideration. Accordingly, the positive third-harmonic component is included in *U_C_*.

The output angle β in the presence of harmonic components is equal to

(15) $$\beta =\arctan\frac{A\sin\theta +Ak_{3}\sin(3\theta )}{A\cos\theta +Ak_{3}\cos(3\theta )}$$

In MATLAB simulations, a fourth-harmonic electrical error is readily seen in the presence of harmonic components. It can also be clearly seen from simulation of Figure 4a that a fourth-order periodic error is caused by the interface between the collection electrodes.

#### 3.1.3. Edge Roughness Error of the Sensitive Electrode

**Figure 5:**
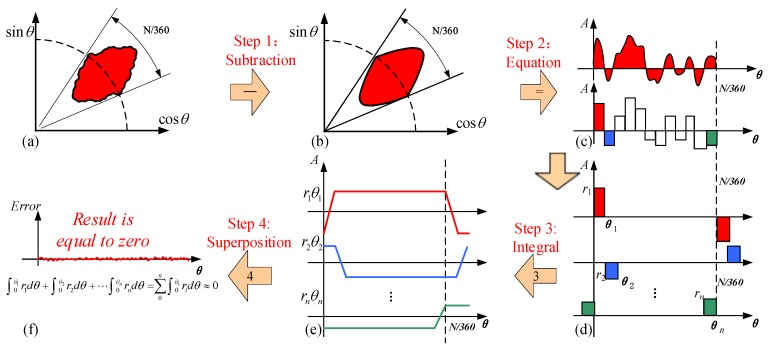
Illustration of an area integral with induction electrodes incorporating random edge roughness. (**a**) Manufactured electrode pattern with edge roughness; (**b**) the electrode area is decomposed into a standard sinusoidal area and an irregular area; (**c**) the irregular area is approximated as a series of small rectangular areas; (**d**) the distinguished small rectangular areas; (**e**) the variation of each rectangular area is obtained through integration; (**f**) the superposition of all trapezoidal waves is expected to approach zero.

The processing error of the rotor is also critical for the measurement accuracy. In this section, the edge roughness error of the sensitive electrode on the rotor is discussed. The analysis of the edge roughness error effect to the nonlinear error is illustrated in Figure 5.

Ideally, the sensitive electrode on the rotor is comprised by a sinusoidal petal-shape as presented in Equation (1). However, the sinusoidal petal-shape is not smooth as a processing error exists. The difference between the irregular area and the standard sinusoidal area is shown in Figure 5a,b, and the residual curve is shown in (c), which can be approximately represented by a group of small rectangular areas (d). The area of each rectangle is obtained as $\int_{0}^{\theta_{i}}r_{i}d\theta$. The areal integral result of the small rectangle is shown in (e). The sum of the areal integral result is shown in (f). Superposition of all rectangular areas is expected to approach zero (Step 4). This operation can be expressed as the following superposition:

(16) $$\Delta S=\int_{0}^{\theta_{1}}r_{1}d\theta +\int_{0}^{\theta_{2}}r_{2}d\theta +\int_{0}^{\theta_{3}}r_{3}d\theta +\cdots \int_{0}^{\theta_{n}}r_{n}d\theta =\sum_{0}^{n}\int_{0}^{\theta_{i}}r_{i}d\theta$$

As shown in Figure 5, theoretical models were established to analyze the smoothing mechanisms of such features in detail, and the effect of the imperfections in the shape of a sinusoidal shaped electrode can be eliminated by applying the integral theorem. Based on this, we conclude that the nonlinear error caused by edge roughness error of the sensitive electrode has little effect on the measurement results as superposition [21].

### 3.2. Circuit Error Analyses

#### 3.2.1. Analysis of Gain and Offset Errors

As the carrier signal sin(wt) will have little impact on the measured angle, the effect of the carrier signal is disregarded on circuit error analyses., and Equation (5) can be simplified as follows,

(17) $$U_{S}=A\sin\theta ,\;U_{S}=A\cos\theta$$

**Figure 6:**
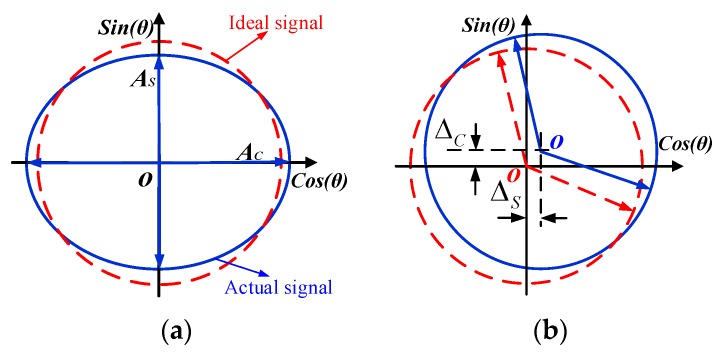
(**a**) Unequal amplitudes of sine and cosine signals; (**b**) sine and cosine signals with offset.

As the result of mismatch errors in the circuit, the final calculated signal will not be a perfect triangular signal [22,23]. Such mismatch is primarily induced by charge and differential amplifier parameters. Figure 6a shows the gain error of the quadrature signal. Ideally, the amplitudes of $U_{S}$ and $U_{C}$ will be equal, and their Lissajous figure should be a circle. Mainly due to the mismatch of the differential amplifier parameters, the Lissajous figure becomes an ellipse whose long axis is $A_{C}$ and minor axis is $A_{S}$. Figure 6b shows the offset error of the quadrature signal due to the signals that have direct current components $\Delta_{C}$ and $\Delta_{s}$ [24,25].

In the case of gain and offset errors, the sinusoidal encoder signals are given by

(18) $$U_{S}=A_{S}\cdot \sin\theta +\Delta_{s},\;U_{C}=A_{C}\cdot \cos\theta +\Delta_{c}$$

When $A_{S}=A_{C}=A$ and $\Delta_{S}=\Delta_{C}=0$, we obtain the ideal amplitude modulated signals.

From the formula $a\sin t+b\cos t=\sqrt{a^{2}+b^{2}}\sin(t+\arctan(b/a))$, the output angle after demodulation can be expressed as Equation (19) when the gain error exists,

(19) $$\beta =\arctan\frac{A_{S}\sin\theta }{A_{C}\cos\theta }$$

Similarly, in the presence of offset error the output angle β is equal to

(20) $$\beta =\arctan\frac{\left(\sin\theta +\Delta_{S}\right)}{\left(\cos\theta +\Delta_{C}\right)}$$

Simulation in MATLAB reveals obvious first- and second-harmonic electrical errors in the presence of offset and gain error, respectively. In the signal processing circuit, C-V conversion is achieved through the use of quad rail-to-rail input and output by single-supply amplifiers. The C-V converters are the key modules in the analog signal processing component; in fact, the main nonlinear error shown in Figure 2b is affected by the mismatch of the feedback capacitor on the C-V converter modules. This mismatch causes a first-harmonic error in the electrical period and is hard to eliminate; fortunately, the first-harmonic error is repeated and the periodic can therefore be compensated for by applying the harmonic wave compensation method.

#### 3.2.2. Analysis of Phase Error

In addition to the effect of gain and offset errors, the phase difference between two standard amplitude modulation signals can also have a significant effect on the output angle, which requires strict orthogonality in the time domain. While signals are non-orthogonal, the two amplitude-modulated signals can be completely expressed as

(21) $$U_{S}=A\sin(\theta +\varphi ),\;U_{C}=A\cos\theta$$

In Equation (21), the amplitudes of the modulation signals are equivalent and φ is the non-orthogonal error in the time domain. Non-orthogonality is mainly caused by the analog signal processing circuit, which contains C-V converters, differential amplifiers, and band pass filters. In a manner similar to that applied in the demodulation procedure, the output angle β corresponding to the output signal in Equation (21) can be obtained as

(22) $$\beta =\arctan\frac{A_{2}^{\prime }\sin\theta_{1}}{A_{2}^{\prime }\cos\theta_{1}-A\sin\varphi \sin\theta }$$

where $A_{2}^{\prime }=\sqrt{[{\left(A\cos\varphi \right)}^{2}+A^{2}]/2+[{\left(A\cos\varphi \right)}^{2}-A^{2}]\cos2\theta /2}$ and $\theta_{1}=\arctan(\sin\theta /\cos\varphi \cos\theta )$.

MATLAB simulation results reveal a clear second-harmonic electrical error and a DC error in the presence of phase error.

### 3.3. Installation Error Analyses

**Figure 7:**
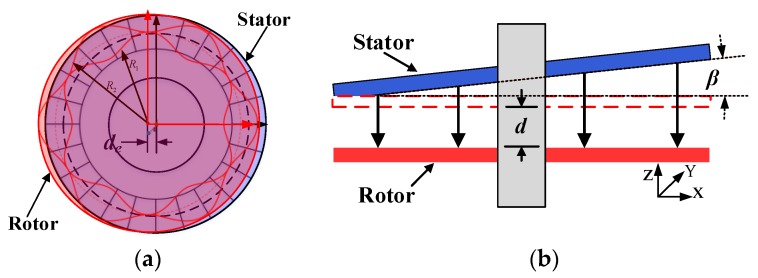
Schematic of installation errors. (**a**) Eccentricity between rotor and stator; (**b**) tilt error of rotor and stator.

Installation error analysis of the angle encoder is important and unavoidable. According to the sensing principle, measurement accuracy is sensitive to two misalignments: eccentricity between rotor and stator, and rotation around the spindle-axis [26]. Figure 7a illustrates rotor eccentricity with respect to the stator with a displacement *d_e_*. Figure 7b shows a rotor rotated around the x-axis relative to the stator, where *β* is the rotational angle and *d* is the width of the average gap between the stator and rotor. The parametric sweep function of COMSOL software was used to investigate the capacitance changes in the amplitude modulation signals at different tilt and eccentricity conditions. To reduce the amount of simulation data required, we did not consider the impact of the excitation or coupling electrodes. In the installation error simulation, it was assumed that there was a stable carrier voltage signal on the sensing electrode.

**Figure 8:**
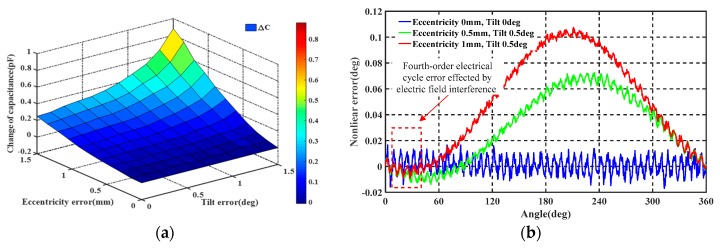
(**a**) Capacitance change at various tilts and eccentricities; (**b**) Angular nonlinear error caused by eccentricity and inclination.

The simulation results of capacitance change are shown in Figure 8a, from which it is seen that neither changing $d_{e}$ from 0 to 0.5 cm nor changing β from 0 to 0.5 deg has a significant effect on the output capacitance, indicating that the encoder is robust to mounting errors over these ranges. This suggests that, if the installation of eccentricity tolerance is no greater than 0.1 mm, the installation error can be ignored. A comparison of the disaggregated results suggests that eccentricity has a possibly greater effect on measured capacitance than tilt, indicating that the installation error of the eccentricity is much more sensitive to the tilt of the encoder.

The MATLAB Simulink tools were then used to further study the angular output errors in terms of eccentricity and inclination over the full mechanical period, with the results shown in Figure 8b. The simulation revealed that both eccentricity and inclination cause errors related to the mechanical period and, as shown in the dashed box in the figure, the fourth-harmonic error appears within each electrical periodic. These errors are mainly caused by the spatial effects of the electric field on the four collection electrodes.

**Figure 9:**
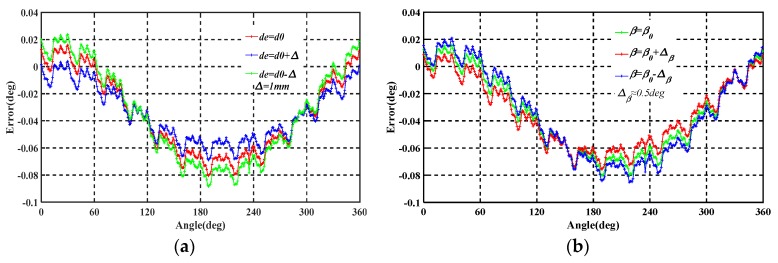
(**a**) Measurement error for different eccentric distances $d_{e}$; (**b**) Measurement error for different tilt angles β.

To further analyze the source of periodic mechanical error, we adjusted the installation and measured the error, which is indicated by the green error curves in Figure 9a,b. Here, the peak-to-peak value changes from 0.1 to 0.08° and the second- and fourth-harmonic components are both slightly reduced.

These errors primarily indicate that both eccentricity and inclination can produce a mechanical periodic error with an amplitude proportional to the eccentricity and inclination. However, a lack of precision measuring equipment made it difficult for us to obtain precise values of eccentricity or tilt, and therefore the corresponding relationships were not analyzed.

## 4. Nonlinear Error Analysis Conclusion

We quantitatively analyzed periodic electrical and mechanical errors using a mathematical error model and simulation, respectively. Table 1 shows the results of the theoretical and installation error analyses and the respective main periodic harmonic components involved. The analyses enabled the tracing of the causes of the first-, second-, and fourth-harmonic periodic electrical errors back to the misalignment of circuit parameters, the fringe effect around the edges of electrodes, and cross interference in the electric field, respectively.

According to the analysis and the demodulation algorithm, the real output angle $\theta_{R}$ can be expressed by the following formula.

(23) $$\theta_{R}=\theta +E_{dc}+A_{0}\cos(\phi +\beta_{0})+A_{1}\cos(\theta_{T}+\beta_{1})+A_{2}\cos(2\theta_{T}+\beta_{2})+A_{3}\cos(4\theta_{T}+\beta_{3})$$

where θ is the true electrical period angle and $E_{dc}$ is the error in the DC component, which is caused by the phase error of the quadrature signals and installation errors such as tilt and eccentricity. $A_{0}\cos(\phi +\beta_{0})$ is the mechanical period error, which is caused by the installation error including the tilt and eccentricity. $A_{1}\cos(\theta_{T}+\beta_{1})$ is the first-harmonic electrical periodic error, which is caused by the offset error on the C-V module. $A_{2}\cos(2\theta_{T}+\beta_{2})$ is the second-harmonic periodic electrical error, which is caused by the gain error in the differential amplifier, the phase difference error, and the non-consistent segmentation error in the collection electrodes. $A_{3}\cos(4\theta_{T}+\beta_{3})$ is the fourth-harmonic periodic electrical error, which is mainly caused by the harmonic interference of the four collection electrodes. This error spectrum analysis reveals the causes of nonlinear error and how its effects can be gradually reduced. An understanding of these cause-and-effect relationships, together with an application of Equation (23), provides a good theoretical underpinning and resource for optimizing the design of a single-excited petal-shaped capacitive rotary encoder through the use of the harmonic compensation method.

## 5. Experimental Setup, Measurement, and Compensation

### 5.1. Experimental Setup and Prototype

**Figure 10:**
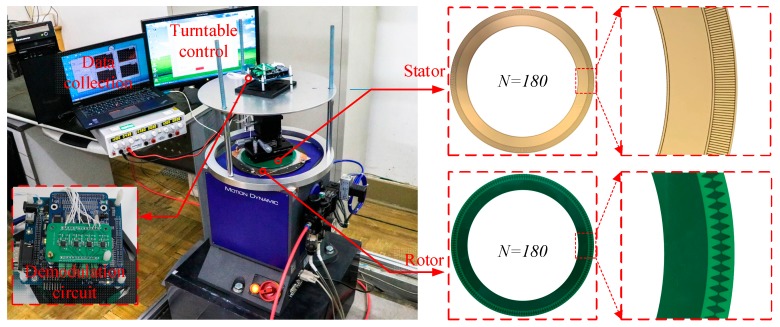
Experimental setup comprising the high-precision air-floating turntable (TES-3V_AB, MOTION DYNAMIC Co., AU) with positional accuracy up to ±0.8 arcsec, Other devices include a stator, rotor, demodulation circuit, data acquisition computer, and turntable control computer. The number of prototype petal-shapes is 180.

To test and compensate a capacitive angular sensor, an experimental system was built. The experimental setup, shown in Figure 10, included a high-precision air-bearing turntable TES-3V_AB (MOTION DYNAMIC Co., AU). The main parameters of the turntable, which had a positioning accuracy of better than ±0.8 arcsec (peak to peak), are shown in Table 2. Other devices included a stator, rotor, demodulation circuit, data acquisition computer, and turntable control computer.

The experiments were conducted on a five-axis precision stage to which the stator was attached to enable adjustments for reducing the concentricity and tilt error between the stator and rotor. The high-precision turntable measured the angular position of the rotor as a reference. When the rotor stopped at a specific angle, the capacitive rotary encoder output would stop at another angle: comparison of the two output angles enabled the curve of the nonlinear error to be obtained.

A capacitive rotary encoder with 180 petal shapes was designed and manufactured. The encoder had inner and outer dimensions of 116 and 148 mm, respectively. Based on the error analysis shown in Section 3, the encoder was optimized as follows.

The encoder structure was fabricated using a high-precision printed circuit board process with a manufacturing precision of 1 mil. The stator and rotor were fabricated with a routing width of 4 mil a clearance of 4 mil, a via hole size of 6 mil, an impedance control tolerance of ± 10%, and a mechanical dimensional tolerance of 4 mil. Based on the theoretical analysis, the edge consistency of the splitting could be attributed to the averaging effect. High precision or low precision machining did not affect the measurement results. The structure of the encoder is shown in Figure 10. The key parameters of the capacitance angular encoder and manufacturing parameters are shown in Table 3.

The demodulation circuit was improved. The preceding circuit error analysis revealed that offset, amplitude, and phase errors arise in the C-V converters, differential amplifier, and filter, respectively. In the most critical part, the C-V converter module, the trace length of the sensitive capacitor was shortened to reduce the parasitic capacitance. The four-way feedback capacitors were carefully selected to ensure equivalent capacitance values. Sine and cosine amplitude matching were achieved using an adjustable precision resistor. To reduce the phase error, the circuit was designed to be as symmetric as possible to ensure that the two signals were orthogonal.

### 5.2. Prototype Measurement

#### 5.2.1. Nonlinear Error Test

**Figure 11:**
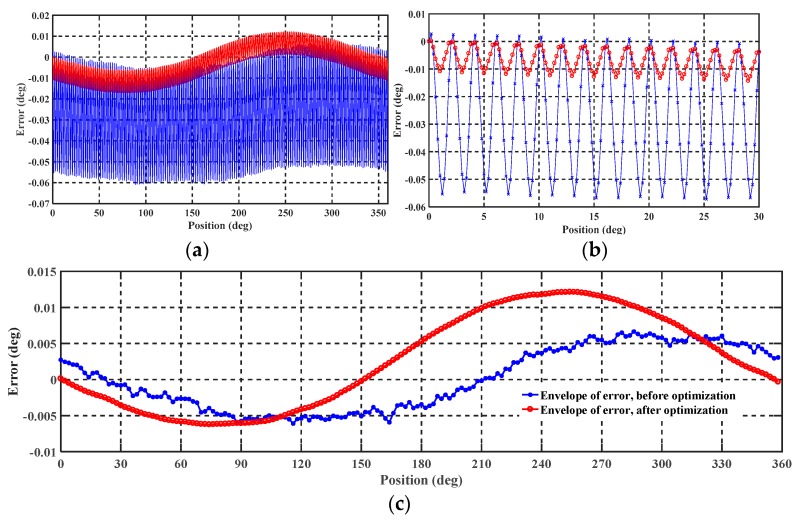
Nonlinear error before and after improvement (especially on demodulation circuit and structure fabricating technology). (**a**) Nonlinear error over the full mechanical period from 0 to 360°; (**b**) nonlinear error over 15 electrical periods from 0 to 30°; (**c**) envelope of nonlinear error before and after improvement.

Based on the above experiment setup, the nonlinear error of the prototype before and after improvement was tested and is shown in Figure 11. The 10 points over an electronic period were selected, and there were 1800 measured data points in the mechanical period. The envelope curve of the nonlinear error with an interval of 2° is plotted in Figure 11c.

The blue and red curves in Figure 11a show the test results before and after improvement, respectively. It is seen that the primary nonlinear error is the first-harmonic electrical error, which is caused by mismatch of the four feedback capacitors applied on the C-V conversion modules. The mismatch causes the DC component, *C*_0_, in Equation (2) to be difficult to eliminate. After optimization and improvement, the peak-to-peak value of the first-order nonlinear error is significantly reduced from 0.08° to 0.02° as shown in Figure 11b. Further, it can also be seen from Figure 11b,c that the consistency and repeatability of the nonlinear error in the electrical periodic are both significantly reduced and the envelope of nonlinear error has become very smooth. These characteristics indicate that the improved sensor can more easily achieve high-precision angular measurement through compensation.

#### 5.2.2. Repeatability Test

**Figure 12:**
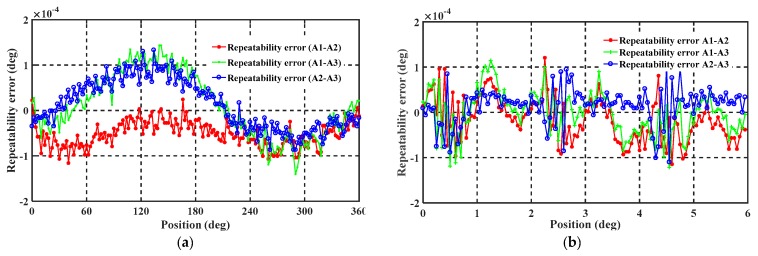
Repeatability test result: (**a**) repeatability error over one mechanical periodic (0–360°) and (**b**) repeatability error over three electrical periodics (0–6°).

Repeatability experiments of the encoder were conducted over three repetitions with a measuring range of 0 to 360°. As shown in Figure 12a, the obtained results vary within ±0.00015°, with a mean standard deviation of ±0.00005°. Similarly, the repeatability experiments were tested with three electrical periodics with a measuring range of 0 to 6°, the results are shown in Figure 12b. These vary within ±0.0001°, with a mean standard deviation of ±0.00005°. It is seen from the figure that the repeat performance is excellent, with repeatability of no more than ±0.00015° and approximately within ±0.0001° over ranges of 0 to 360° and 0 to 6°, respectively. The repeatability errors are mainly due to the measurement system and environmental interference. Excellent repeatability levels indicate that this single-excitation petal-shape sensor has the potential to achieve high-precision measurements after compensation.

### 5.3. Nonlinear Error Compensation

#### 5.3.1. Compensation for Mechanical Periodic Error and DC Errors

**Figure 13:**
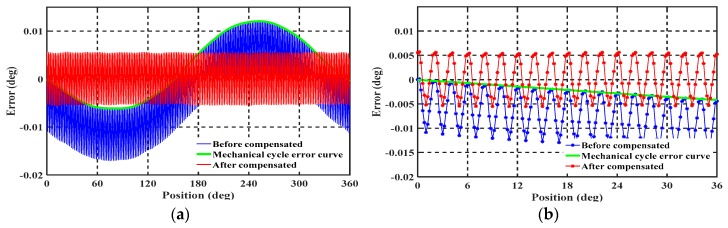
Diagram of nonlinear error before and after compensation of the mechanical periodic error. (**a**) Measurement error over the full measurement range from 0 to 360°; (**b**) Measurement error over 18 electrical periods from 0 to 36°.

The above repeatability test shows that the single-excitation petal-shaped capacitive rotary encoder has good repeatability over electrical and mechanical ranges that do not exceed 0.0003 deg over 0–360 deg. The compensation method was applied to further reduce the nonlinear error of the sensor based on optimization [27,28]. The diagram of the nonlinear error before and after compensation of the mechanical periodic error is shown in Figure 13.

According to the error mechanism of the capacitive sensor analyzed in Section 3, the following angular position error model was established to compensate DC error and mechanical period error.

(24) $$f{\left(\phi \right)}_{\phi }=d_{\phi }+A_{\phi 1}\cos(\phi +\beta_{\phi 1})+A_{\phi 2}\cos(2\cdot \phi +\beta_{\phi 2})+A_{\phi 3}\cos(3\cdot \phi +\beta_{\phi 3})$$

where $d_{\phi }+A_{\phi 1}\cos(\phi +\beta_{\phi 1})$ is caused by installation error. $A_{\phi 2}\cos(2\cdot \phi +\beta_{\phi 2})+A_{\phi 3}\cos(3\cdot \phi +\beta_{\phi 3})$ is the higher harmonic component of the mechanical periodic error, the component value is small, and can be ignored in general [23]. The coefficient vector in the model is defined as $C_{\phi }=[d_{\phi },A_{\phi 1},\beta_{\phi 1},A_{\phi 2},\beta_{\phi 2},A_{\phi 3},\beta_{\phi 3}]$.

The least-squares function of the MATLAB software was used to fit the angular position nonlinear error curve and obtain the model coefficient vector:

(25) $$C_{\phi }=[0.0055,0.0093,-2.9560,0.0039,0.2108,0.000076,0.5305]$$

**Figure 14:**
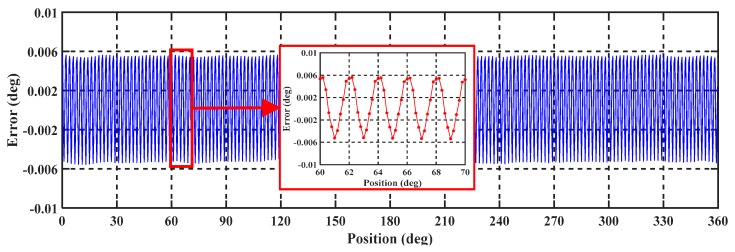
Nonlinear error test results following compensating for mechanical periodic error. The nonlinear error after compensation is less than 0.001° over the full mechanical periodic and within the electrical periodic. The error repeatability is excellent over all the electrical angle period.

The model was then used to compensate the output angle signal of the angular displacement sensor and the mechanical period error. The compensated error curve is shown in Figure 14, from which it is seen that the peak-to-peak errors of the angular position before and after compensation are 0.03° and 0.011°, respectively, and the angular position error after compensation is reduced to half of its original value. The primary purpose of the mechanical periodic error compensation was to make the error within the electrical period reproducible, which is conducive to subsequent compensation of the electrical periodic error.

The angular output following the compensation of the mechanical periodic and DC errors is shown in Figure 14, from which it is seen that the nonlinear error after compensation is less than 0.001° over the full mechanical periodic. Furthermore, the error repeatability is excellent within the electrical angular period.

#### 5.3.2. Compensation for Electrical Periodic Error

Finally, we provided further compensation for the electrical periodic error. The preceding analysis revealed that the primary errors in the electrical periodic are first-, second-, and fourth-order errors; the corresponding error model for harmonic compensation can thus be expressed as:

(26) $$f{\left(\phi \right)}_{\theta }=d_{\theta }+A_{\theta 1}\cos(\theta_{T}+\beta_{\theta 1})+A_{\theta 2}\cos(2\theta_{T}+\beta_{\theta 2})+A_{\theta 3}\cos(4\theta_{T}+\beta_{\theta 3})$$

The coefficient vector in the model is defined as *C_θ_* = [*d_θ_*, *A_θ_*_1_, *β_θ_*_1_, *A_θ_*_2_, *β_θ_*_2_, *A_θ_*_3_, *β_θ_*_3_].

The same method was used to fit the electrical period error model and obtain the coefficient vector values

(27) $$C_{\theta }=[0.0002,0.0063,1.4737,4.2281^{-4},-0.9743,4.7639^{-4},4.0053]$$

**Figure 15:**
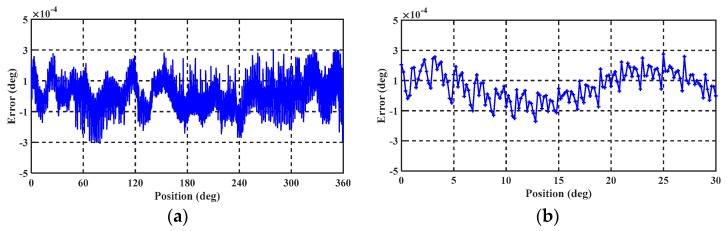
(**a**) Nonlinear error from 0 to 360° following electrical periodic error compensation; (**b**) nonlinear error from 0 to 30° following electrical periodic error compensation.

**Figure 16:**
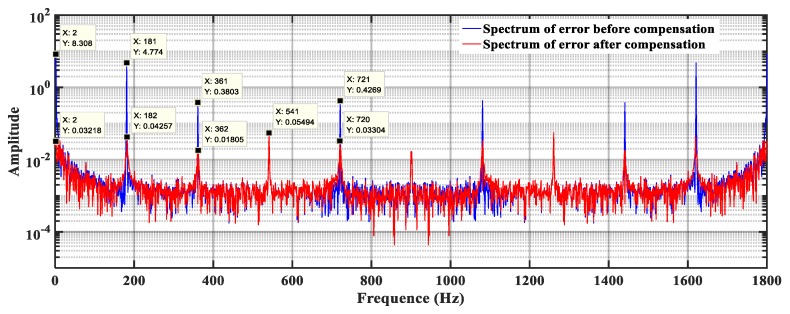
Nonlinear error spectrum distribution before and after harmonic compensation. The DC component, mechanical periodic error, and frequency component of the electrical periodic error are all significantly reduced following compensation.

The nonlinear error after compensating the electric period error is shown in Figure 15a,b. The angular position error after compensation is 0.0006° which is about 5% of that before compensation. The Fourier transform of the nonlinearity error before and after the compensation was conducted, and the result is shown in Figure 16. It can be seen from the figure that the DC component, the mechanical periodic error, and the frequency component of the electrical periodic error are significantly reduced after the compensation.

The excellent periodic characteristics of the nonlinear error suggested the implementation of a harmonic compensation method that could effectively reduce the nonlinear measurement error to ±0.0003° by compensating for the DC, mechanical periodic, and electrical periodic error. The result of the nonlinear error after compensation is shown in Figure 15. The nonlinear error spectrum distribution before and after harmonic compensation is shown in Figure 16. The DC component, mechanical periodic error, and frequency component of the electrical periodic error are all significantly reduced after compensation.

## 6. Conclusions

In this study, the nonlinear error sources of a single-excited petal-shaped capacitive rotary encoder were theoretically analyzed, with the results showing that the nonlinear sensing error has good periodic characteristics. Analytical and simulation results confirmed that the first-, second-, and fourth-periodic electrical errors are caused by the misalignment of circuit parameters, non-uniform segmentation of the processing error, and cross interference of the electric field, respectively. Further experimental investigation revealed that the mechanical periodic error is caused by installation misalignment. Based on these results, the nonlinear error can be suppressed and optimized from the source. Following optimization, the original sensor nonlinear error was reduced from 0.08° to 0.02° when the number of petal shapes is 180. Based on theoretical analysis of the period nonlinear error, a multi-harmonic angular position error model of the capacitive encoder was established and used to compensate the systemic error of the encoder. Following harmonic compensation and optimization, the nonlinear error of the capacitive sensor was reduced from 0.02° to 0.0006°. The presented study is expected to enable the transformation of the presently challenging technology of single-excited petal-shaped capacitive rotary encoder measurements with arc-second accuracy to a conventional technology.

## Figures and Tables

**Table 1:** Summary of causes of nonlinear error components at various frequencies.

Error Source	Main Harmonic Components	Symbol
Gain error	Second harmonic component	2*θ*
Offset errors	First harmonic component	*θ*
Phase difference error	DC and Second harmonic component	*DC* + 2*θ*
Harmonic components	Fourth harmonic component	4*θ*
Eccentricity error	DC and Mechanical periodic	*DC* + *ϕ*
Tilt error	DC and Mechanical periodic	*DC* + *ϕ*
Non-consistent segmentation error	Second harmonic	2*θ*

**Table 2:** Parameters of the turntable.

Parameter	Value
Position range	0 to 359.999 deg unlimited rotation
Positional resolution	<0.02 arcsec
Positional accuracy	± <0.8 arcsec _peak_peak_
Positional repeatability	Better ± 0.5 arcsec
Axis wobble	±0.5 arcsec
Rate stability	0.001% of commanded rate over 360 deg

**Table 3:** The key parameters of the capacitance rotary encoder and manufacturing parameters.

Parameter (Symbol)	Value	Parameter (Symbol)	Value
Outer radius	74 mm	Mechanical tolerance	4 mil (101.6 µm)
Inner radius	58 mm	Manufacturing precision	1 mil (25.4 µm)
Number of petal-shapes (*N*)	180	Width of the routing	4 mil (101.6 µm)
Distance of stator and rotor (d)	0.5 mm	Via hole size	6 mil (152.4 µm)
